# Evaluation of GPT-4 concordance with north American spine society guidelines for lumbar fusion surgery

**DOI:** 10.1016/j.xnsj.2024.100580

**Published:** 2024-12-27

**Authors:** Ara Khoylyan, Jason Salvato, Frank Vazquez, Mina Girgis, Alex Tang, Tan Chen

**Affiliations:** a Geisinger Commonwealth School of Medicine, Scranton, PA, United States; b Geisinger Northeast Orthopaedic Surgery Residency, Wilkes-Barre, PA, United States; c Department of Orthopaedic Surgery, Geisinger Medical Center, Danville, PA, United States

**Keywords:** Artificial intelligence, ChatGPT, Lumbar fusion surgery, Degenerative disc disease, NASS guidelines, Large language models

## Abstract

**Background:**

Concordance with evidence-based medicine (EBM) guidelines is associated with improved clinical outcomes in spine surgery. The North American Spine Society (NASS) has published coverage guidelines on indications for lumbar fusion surgery, with a recent survey demonstrating a 60% concordance rate across its members. GPT-4 is a popular deep learning model that receives knowledge training across public databases including those containing EBM guidelines. There is prior research exploring the potential utility of artificial intelligence (AI) software in adherence with spine surgery practices and guidelines, inviting opportunity to further investigate application in the setting of lumbar fusion surgery with current AI models.

**Methods:**

Seventeen well-validated clinical vignettes with specific indications for or against lumbar fusion based on NASS criteria were obtained from a prior published research study. Each case was transcribed into a standardized prompt and entered into GPT-4 to obtain a decision whether fusion is indicated. Interquery reliability was assessed with serial identical queries utilizing the Fleiss’ Kappa statistic. Majority response among serial queries was considered as the final GPT-4 decision. Queries were all entered in separate strings. The investigator entering the prompts was blinded to the NASS-concordant decisions for the cases prior to complete data collection. Decisions by GPT-4 and NASS guidelines were compared with Chi-square analysis.

**Results:**

GPT-4 responses for 15/17 (88.2%) of the clinical vignettes were in concordance with NASS EBM lumbar fusion guidelines. There was a significant association in clinical decision-making when determining indication for spine fusion surgery between GPT-4 and NASS guidelines (χ² = 9.75; p<.01). There was substantial agreement among the sets of responses generated by GPT-4 for each clinical case (K = 0.71; p<.001).

**Conclusions:**

There is significant concordance between GPT-4 responses and NASS EBM indications for lumbar fusion surgery. AI and deep learning models may prove to be an effective adjunct tool for clinical decision-making within modern spine surgery practices.

## Background

There are approximately 500,00 lumbar spine surgeries performed in the United States each year, a significant portion of which are spinal fusions. Between 1998 and 2008, spinal fusions increased in annual incidence by 171% and, since 2004, national expenditure for such has increased 7.9-fold [[1], [2], [3], [4], [5]]. There are several proposed contributing factors to the increased rate of spinal fusions, including an aging U.S. population, improvements in surgical technique and technology leading to improved outcomes, and variability in clinical practice and guidelines [3,5]. This study explores clinical practice and guidelines. By investigating the concordance of emerging artificial intelligence (AI) software with established guidelines, our study intends to explore an opportunity for augmenting clinical practice by decreasing heterogeneity in clinical decision-making for operative care [2,3].

To address inconsistencies in clinical criteria for lumbar fusion surgery, the North American Spine Society (NASS) developed evidence-based medicine (EBM) guidelines for diagnoses, qualifying criteria, and indications for lumbar spinal fusion surgery [6]. These guidelines aim to reduce the heterogeneity of indications and provide optimal patient outcomes [3,[6], [7], [8], [9]]. The significance of clinical guidelines has gained prominence in guiding surgeons through their decision-making process as evidence has shown that the use of EBM guidelines and algorithms produces improved clinical and functional outcomes [3,[10], [11], [12]]. In fact, EBM concordant patients who underwent lumbar fusion surgery demonstrated improved outcomes by a factor of 3 compared to those not meeting EBM lumbar spinal fusion criteria [3]. A recent study surveying North American spine surgeons demonstrated a compliance rate of 60% with NASS guidelines for lumbar fusion surgery [11].

A potential important step in the path toward standardization of care with NASS EBM concordance may be the development and implementation of AI technology. Chat Generative Pre-Trained Transformer-4 (ChatGPT-4 or GPT-4) is a multimodal large language model (LLM) with more than one trillion parameters that is pre-trained on publicly available sources such as published EBM guidelines [13,14]. The software additionally has a built-in internet browsing capability, allowing contextual emergence. Given the extent of background and contextual knowledge, we hypothesize that this tool will have significant concordance with NASS EBM guidelines, though there may be some contention in prior literature. A study from early 2024 demonstrated significant alignment between GPT-3.5 – a predecessor to GPT-4 with less technical capabilities – and NASS Clinical Guidelines for the Diagnosis and Treatment of Degenerative Lumbar Spinal Stenosis [15]. However, other studies in the realm of spine surgery involving various ChatGPT models have demonstrated circumstantial challenges with correctly predicting surgical risks, success rates, selection of surgical approaches, and decisions by NASS guidelines [[16], [17], [18], [19], [20]].

LLMs are not currently used in standard practice to inform surgical decision-making, and our study does not explore the clinical application of GPT-4. However, we do compare GPT-4 decisions for lumbar fusion surgery in real clinical presentations with those of established NASS guidelines and prior surveyed North American spine surgeons. The lack of a strong consensus in prior literature on the utility of GPT-4 adherence to established clinical guidelines for lumbar fusion invites opportunity for further exploration. Given that EBM guideline adherence leads to improved clinical outcomes, we anticipate that an increased relative concordance can strengthen arguments for potential utility of the model as an adjunct clinical tool.

## Methods

The institutional review board exempted the present study from review because the study involved no human participants. The reporting of findings is conducted according to the Transparent reporting of a multivariable prediction model for individual prognosis or diagnosis (TRIPOD) statement and Standards for Reporting of Diagnostic Accuracy Studies (STARD) 2015 guidelines [21,22]. We analyzed a total of 17 thorough, well-validated clinical cases involving lumbar spine etiology at the L2-S1 levels [11]. The L4-5 level was most frequently the primary site of injury or degeneration (41.2%) and spinal stenosis was the most common diagnosis based on imaging (47.1%). The median age of the sample patient population was 57.4 years (Range: 32-83, SD = 15.9). Female patients composed 41% of the population.

**Fig. 1:**
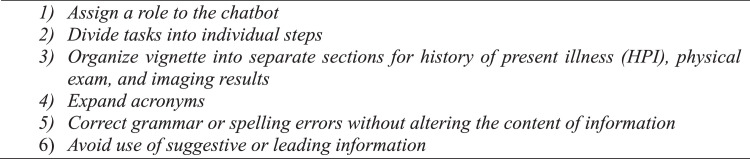
Criteria for prompt design.

We evaluated the software capabilities of an active ChatGPT model [GPT-4 (Dec. 2023 – Sep. 2024)], which can produce natural language text in response to complex scenarios generated by user-based input [14]. This software can be accessed online for free or more readily with a monthly subscription [14]. Each vignette was composed of anonymized patient history of present illness, prior treatment outcomes, physical examination findings, and descriptive imaging results (computerized tomography (CT) scan, magnetic resonance image (MRI), or plain radiograph). Only the descriptive image reports were entered into the standardized prompts due to the software's insufficient image analysis capability. The prompt was designed according to strict predefined criteria (Fig. 1).

**Fig. 2:**
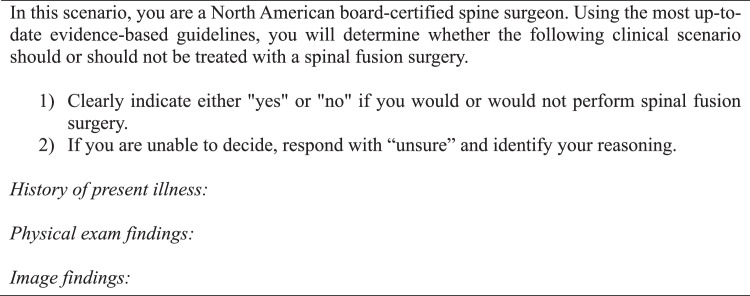
Standardized template utilized for each query.

**Fig. 3:**
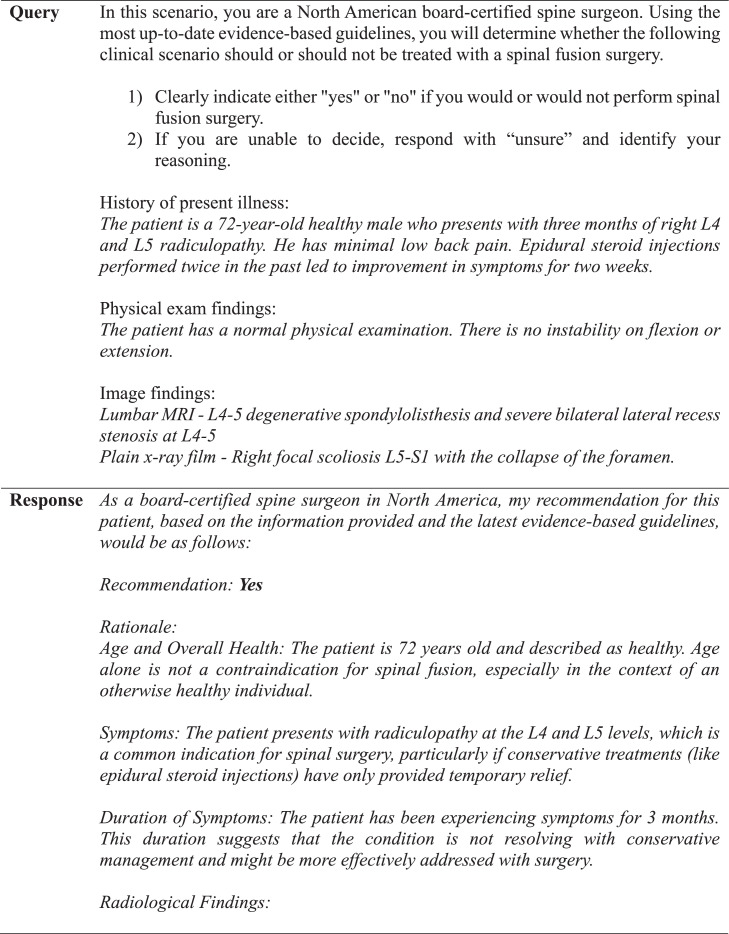

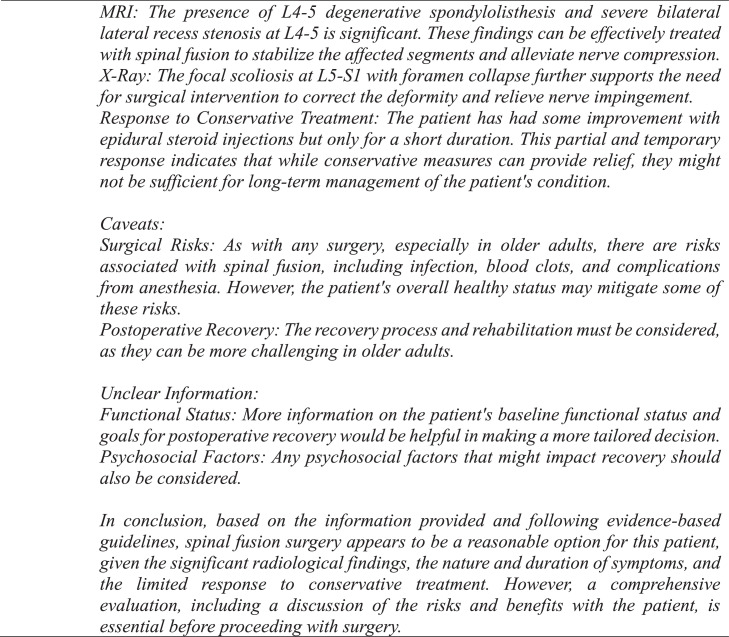
Example of an exchange in which GPT-4 reaches a definitive conclusion for lumbar fusion surgery.

A panel of senior neurosurgeons and spine surgeons had previously framed and discussed each vignette, agreeing that each case had definitive conclusions for or against fusion surgery based on indications drawn from NASS guidelines [6,11]. These conclusions were established as the NASS decisions. For the GPT-4 decisions, a template was created and used to generate a standardized prompt for each clinical case (Figs. 1, 2, 3). One investigator queried GPT-4 with each case prompt 3 times to assess interquery reliability under identical software conditions, which involved accessing GPT-4 in incognito mode on Google Chrome and entering each query into a separate string to negate the effect of the software's limited contextual memory. If there were differences in response between requeries for a given case, the software's decision was determined by majority rule. Responses by GPT-4 were categorized as “yes,” “no,” or “unsure” and subsequently compared with NASS decisions. The investigator who input the prompts into GPT-4 was blinded to the NASS decisions prior to complete data retrieval. No abbreviations were used. Cases were individually reviewed and subsequently tagged with numeric identifiers.

### Prompt design

We provide a template for prompt design that was utilized in our study (Fig. 1), which yielded the substantial observed concordance and predictive capabilities. We mention 6 important features in the design of our prompt, including assignment of an appropriate role to the chatbot, division of tasks into individual steps, organization of vignettes into separate sections based on HPI, physical exam, and imaging, avoidance of acronyms or abbreviations, and use of correct grammar and spelling. These criteria were implemented to maximize clarity in the prompt and help better identify nodes of disagreement. We also found it important to administer serial queries to establish a consensus or majority response, given that GPT-4 did not have perfect internal agreement. Prompting with 3 identical queries per case was sufficient to generate the substantial internal and external agreement that we observed. We turned off the “memory” feature and generated each query response in a separate thread to minimize contextual influence or learning. However, GPT-4 has a 128K token context window, so enabling such features would likely yield more accurate responses over time due to gradual contextual knowledge acquisition.

### Statistical analysis

We calculated precision, recall, and F1 score to assess alignment between the GPT-4-generated treatment options and NASS guidelines. True positive was defined as fusion surgery recommended by both the GPT-4 and NASS, false positive as fusion surgery recommended by GPT-4 but not by NASS, true negative as fusion surgery not recommended by both GPT-4 and NASS, and false negative as fusion surgery not recommended by GPT-4 but recommended by NASS. Chi-square analysis (χ²) was also performed to determine whether there is an association in decision-making for spine fusion surgery between GPT-4 and NASS guidelines. To assess the inter-query reliability of the GPT-4 software, the Fleiss’ kappa statistic (K) was measured due to its application with nominal data and number of raters. P values <.05 were considered statistically significant. All statistical analyses were performed using Statistical Packages for Social Sciences v25 (IBM Corporation, Armonk, New York).

## Results

**Fig. 4:**
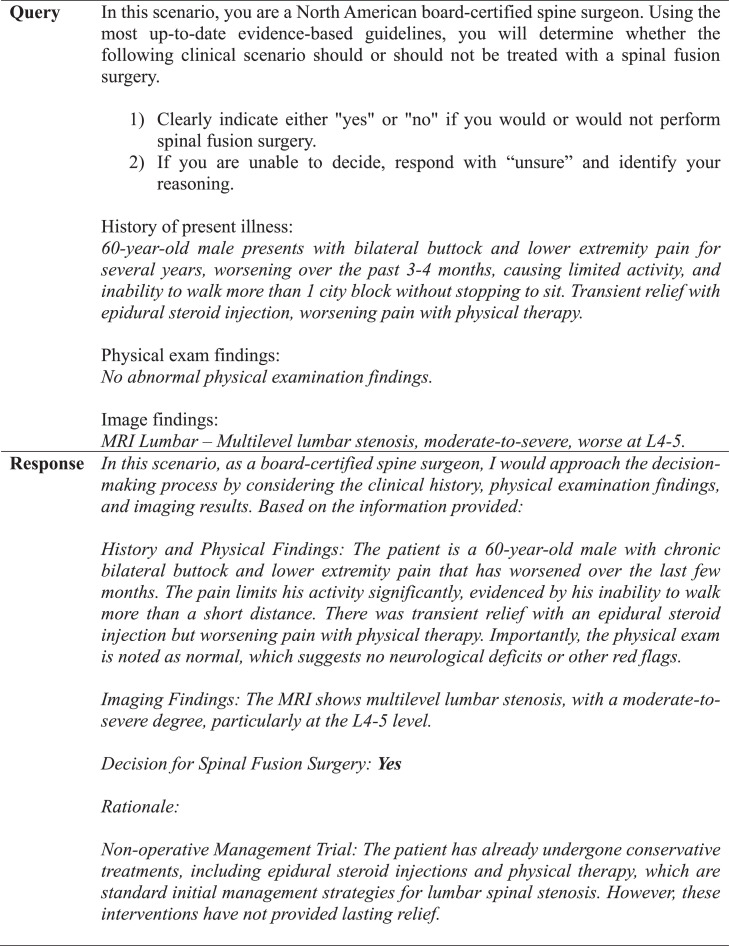

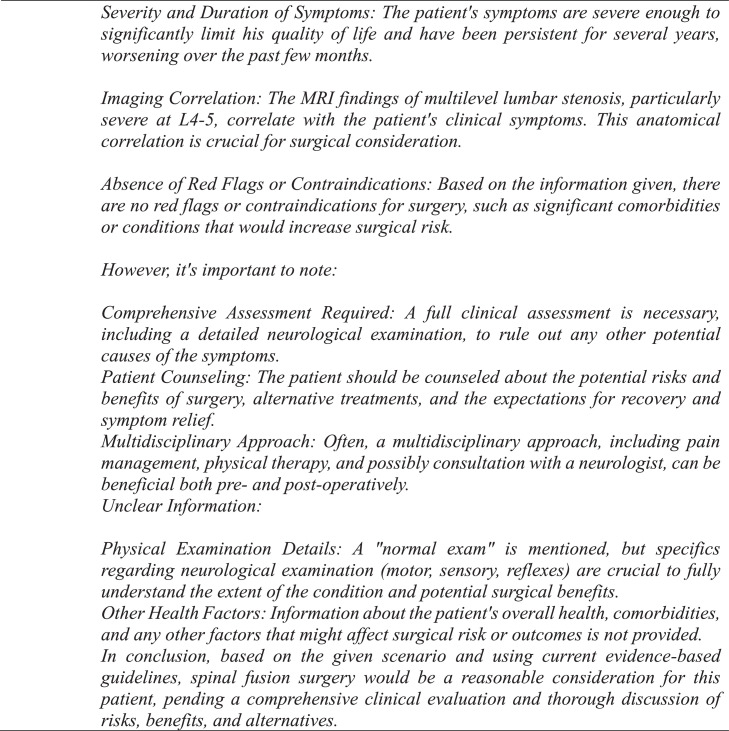
Case involving lumbar stenosis. GPT-4 elects for treatment with lumbar fusion surgery, contradicting decision to manage conservatively based on NASS guideline recommendations.

**Fig. 5:**
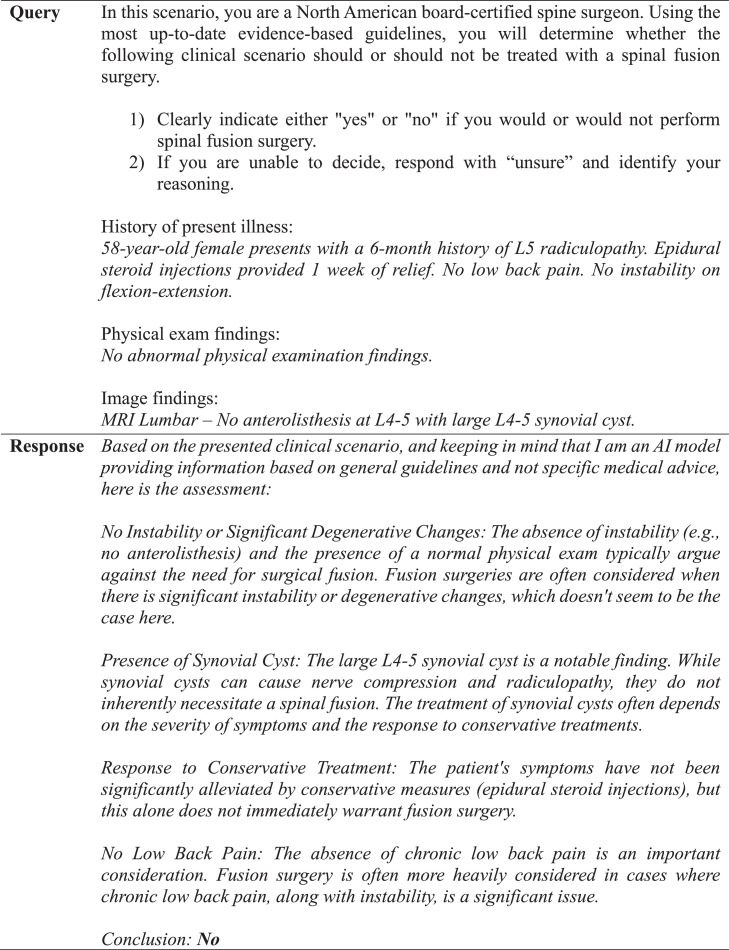

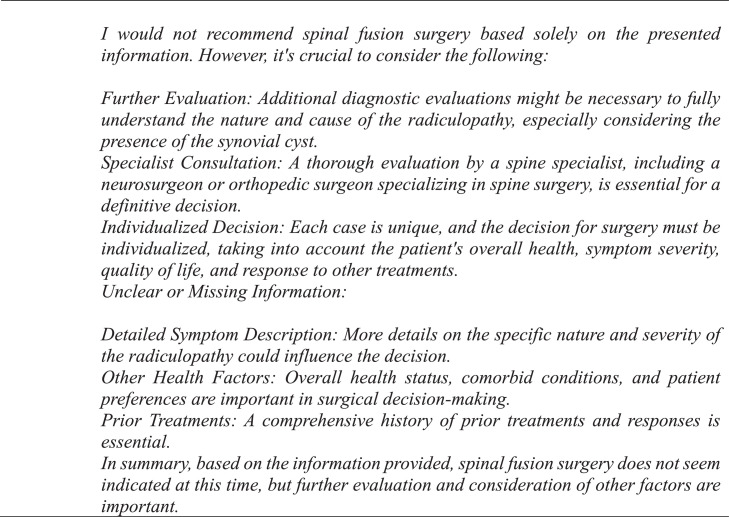
Case involving a synovial cyst. GPT-4 elects against treatment with lumbar fusion surgery, contradicting decision to fuse based on NASS guideline recommendations.

**Fig. 6:**
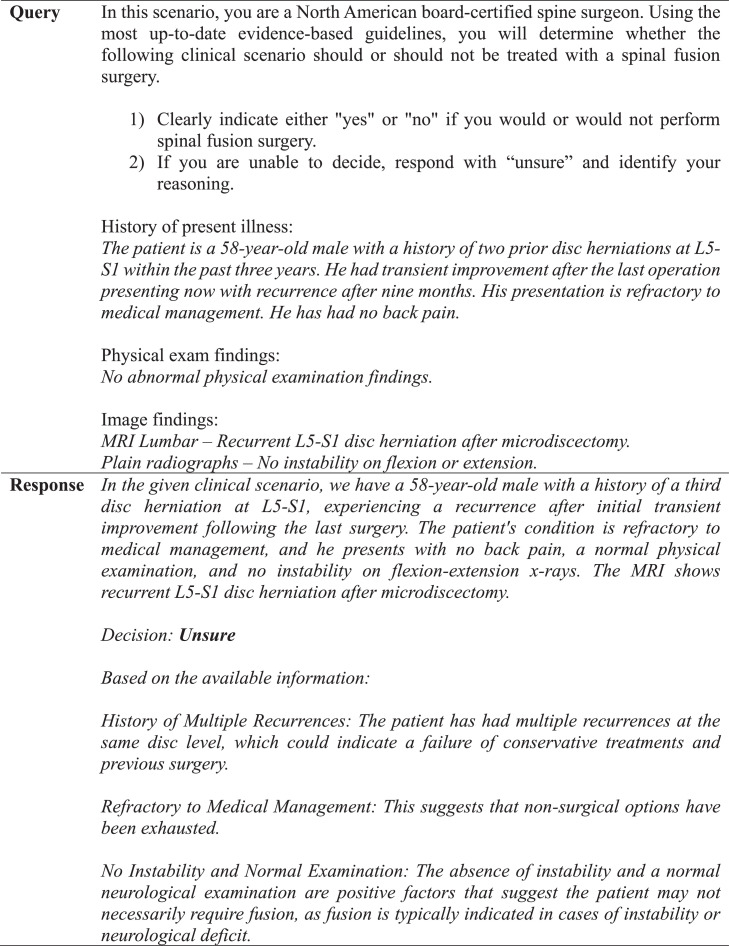

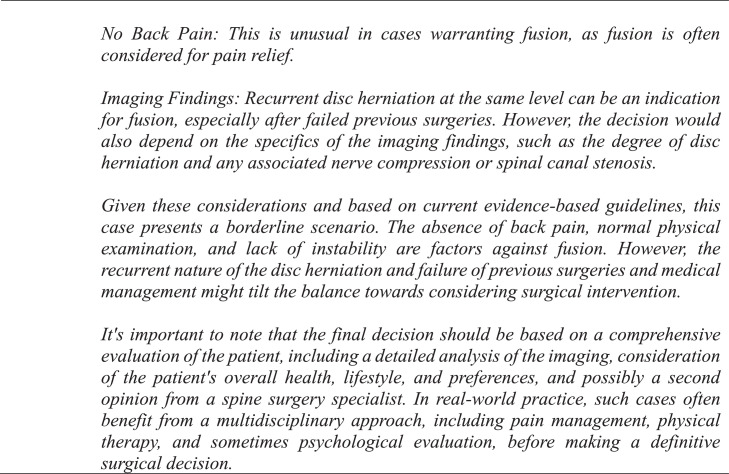
Case involving recurrent disc herniation. GPT-4 is unable to definitively conclude whether lumbar fusion surgery is indicated.

**Fig. 7:**
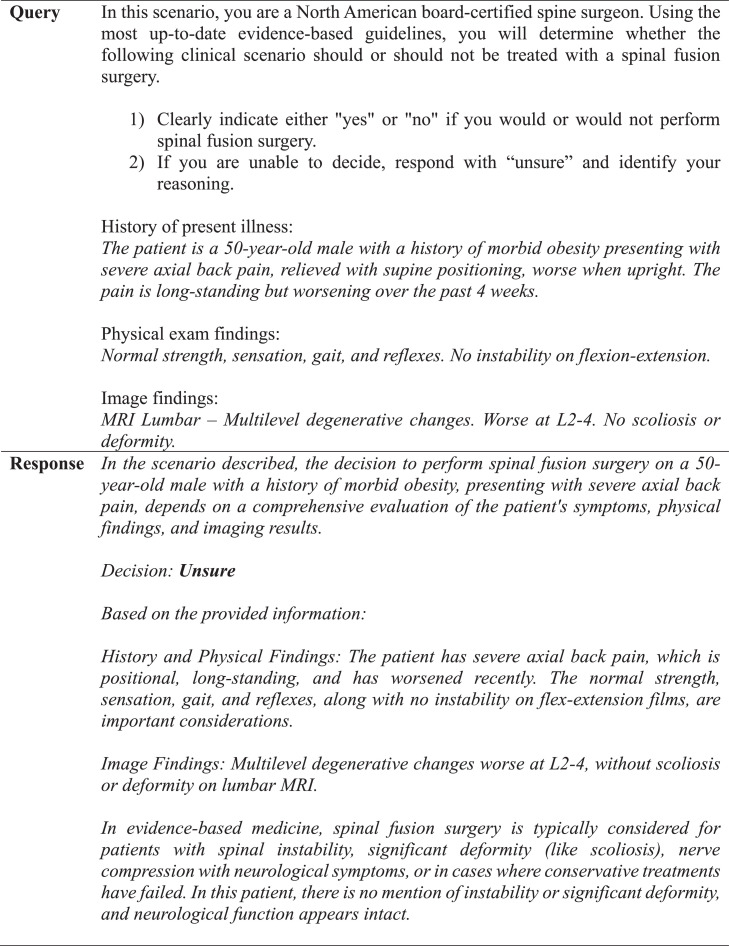

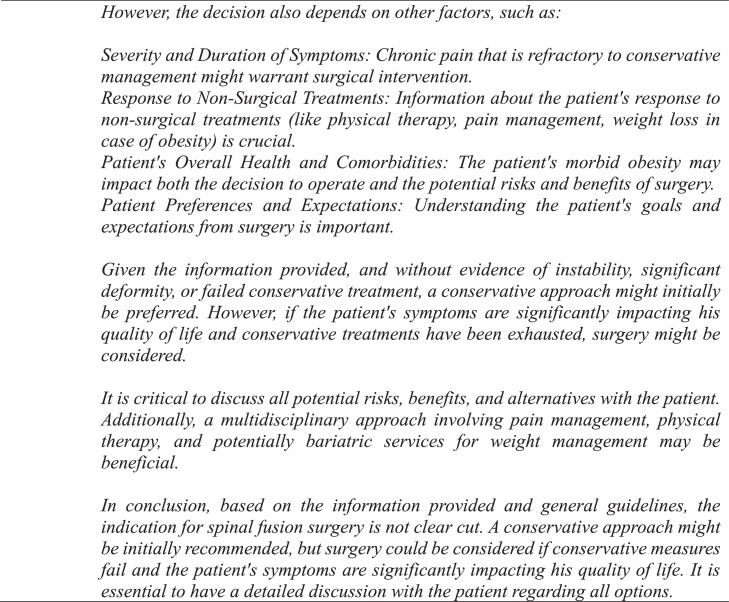
Case involving axial lower back pain. GPT-4 is unable to definitively conclude whether lumbar fusion surgery is indicated.

GPT-4 achieved a precision, recall, and F1 score of 90% (Table 1). The observed concordance rate between GPT-4 and NASS guidelines was significantly higher than the expected concordance rate for both “yes” and “no” responses, indicating a significant association between GPT-4 and NASS guidelines in decision-making for the necessity of lumbar spinal fusion (χ² = 9.75; p<.01) (Table 2). GPT-4 and NASS agreed in fifteen cases (88.2%). In 1 case (5.9%) involving lumbar stenosis, GPT-4 determined that spinal fusion was necessary while NASS indicated that it was not. GPT-4’s rationale for electing for surgery was based on failure of non-operative management, severity and duration of symptoms, imaging correlation, and absence of surgical contraindications (Fig. 4). In one case (5.9%) involving a synovial cyst, GPT-4 determined that spinal fusion was not necessary while NASS determined that it was necessary. The decision for conservative management was based on lack of instability or significant degenerative changes, lack of back pain, and opportunity for further conservative treatments (Fig. 5). GPT-4 was “unsure” in 2/51 (3.9%) queries, which involved recurrent disc herniation and axial lower back pain, respectively (Table 3). In the case involving recurrent disc herniation, GPT-4 found that the history of multiple recurrences and refractory nature to medical management provided reason for fusion surgery though lack of back pain, lack of instability on flexion-extension, and normal physical examination suggested against fusion surgery (Fig. 6). In the case involving axial lower back pain, GPT-4 did suggest that conservative treatment might be favorable but could not definitively conclude this due to lack of information about response to prior conservative management (Fig. 7).

**Table 1:** Overall alignment of GPT-4 decisions with NASS guidelines.

	GPT-4
*Accuracy*	0.88
*Sensitivity (Recall)*	0.90
*Specificity*	0.86
*PPV (Precision)*	0.90
*NPV*	0.86
*F1 Score*	0.90

*PPV, positive predictive value; NPV, negative predictive value.

**Table 2:** Analysis of concordance between GPT-4 with both “Yes” and “No” decisions by NASS guidelines.

GPT-4 decision	Observed % match	Expected % match	Chi-square totals*
*Yes*	90%	59%	1.65
*No*	86%	41%	2.36

⁎ χ^2^ = 9.75, df = 1, p=.002.

**Table 3:** Comparison of GPT-4 and NASS Decisions. Queries 1-3 indicate entries of the same case prompt under identical software conditions for examination of internal reliability.

No.	Case	Level	GPT-4 query 1	GPT-4 query 2	GPT-4 query 3	GPT-4 majority decision	NASS decision
*1*	Adjacent level disease	L3-4	Yes	Yes	Yes	Yes	Yes
*2*	Discitis	L4-5	No	No	No	No	No
*3*	Axial LBP with a trial of nonsurgical therapy	L5-S1	Yes	Yes	Yes	Yes	Yes
*4*	Degenerative Spondylolisthesis	L4-5	No	Yes	No	No	No
*5*	Foraminal stenosis	L5-S1	Yes	Yes	Yes	Yes	Yes
*6*	Recurrent stenosis	L4-5	Yes	Yes	Yes	Yes	Yes
*7*	Recurrent disc herniation	L5-S1	Yes	Unsure	Yes	Yes	Yes
*8*	Degenerative Spondylolisthesis	L4-5	Yes	Yes	No	Yes	Yes
*9*	Axial LBP without a trial of nonsurgical therapy	L2-4	No	Unsure	No	No	No
*10*	Synovial cyst	L4-5	No	No	No	No	Yes*
*11*	Pseudarthrosis	L4-5	Yes	Yes	Yes	Yes	Yes
*12*	Deformity	L3-4	Yes	Yes	Yes	Yes	Yes
*13*	Burst fracture	L2	Yes	Yes	Yes	Yes	Yes
*14*	Deformity and no physical therapy	L3-4	No	No	No	No	No
*15*	Lumbar stenosis	L4-5	Yes	Yes	Yes	Yes	No*
*16*	Transverse process fracture	L2-4	No	No	No	No	No
*17*	Axial LBP	L5-S1	No	No	No	No	No

Note: LBP, Lower back pain.

⁎ Discordance between GPT-4 and NASS.

In four cases (23.5%), GPT-4 had internal disagreement between the initial query and requeries. However, in total, there was substantial internal agreement among the sets of queries generated by GPT-4 for the clinical cases (K = 0.71; p<.001; Percent Agreement = 92.2%). There was greater agreement among “Yes” responses than “No” (K = 0.84 vs 0.68) (Table 4).

**Table 4:** Kappa interquery reliability of GPT-4 responses (evaluating 3 identical queries per case).

	Kappa	Standard error	p-value
*Overall agreement*	.705	.126	<.001
*Yes*	.843	.140	<.001
*No*	.680	.140	<.001
*Unsure*	-.041	.140	.771

## Discussion

The utility of AI models in healthcare is being explored at an accelerating rate as new technologies that support such implementation are being introduced. GPT-4, one of the active models for ChatGPT, has shown to have significant promise in healthcare applications for both increasing efficiency and efficacy of work [23,24]. Given this, some have proposed GPT-4 as a potential guiding element in surgical planning, decision-making, and patient interface for spine surgeons [25]. In our study, we evaluate the utility of GPT-4 with reference to adherence to NASS EBM guidelines.

### GPT-4 concordance with NASS guidelines for lumbar fusion

A previous study surveying 70 AO Spine North America members identified spine surgeons as being “NASS concordant” if their recommendations for lumbar spine fusion surgery were in line with NASS guidelines in ≥70% of surveyed cases [11]. In our study involving the identical clinical cases, we demonstrated that GPT-4 aligned with NASS guidelines in 88.2% of cases when determining indication for lumbar spine fusion, establishing it as NASS concordant based on this previously established parameter. The aforementioned study found that 62.8% of all surgeons surveyed fell in the NASS concordant category. On average, they were concordant with NASS guidelines 72.6% (±11.2%) of the time based on the given clinical scenarios [11]. We found that GPT-4 has 90% precision, recall, and F1 score with reference to predicting NASS decisions. Based on these findings, we demonstrated that GPT-4 is concordant with NASS EBM guidelines to a greater extent when evaluating indication for lumbar spine fusion than the majority of the surveyed board-certified North American spine surgeons. The referenced survey did not ask for responses specifically based on NASS guideline recommendations from the surgeons. Similarly, our prompt for GPT-4 did not mention NASS guidelines, though we recommended adherence to the most up-to-date EBM guidelines.

In cases that GPT-4 was not in agreement with the NASS decision, it was able to provide rationale for discordance. For example, in a case involving a synovial cyst (Fig. 5), it disagreed with NASS guidelines and deemed that surgical fusion was unnecessary due to a lack of instability or significant degenerative changes, lack of back pain, and opportunity for further conservative management. However, it also clarified that it needed further information to make a more informed decision, including details about severity of the radiculopathy, other health factors such as comorbid conditions, and a comprehensive list of prior treatments (Fig. 5). These features demonstrate that utility is not limited to binary decision-making – clinicians utilizing the tool may readily identify and supplement gaps to improve the quality and depth of responses.

These findings are not meant to discount the value of decision-making based on evidence from clinical practice, which many of the surveyed spine surgeons who did not primarily rely on NASS guidelines indicated to be their primary source of guidance. Still, prior research demonstrates that guideline-adherent clinicians yield patients with better outcomes, including post-surgical ODI scores significantly greater than nonadherent clinicians (ODI Score 8 vs 2.1; p=.002)[12]. EBM guidelines ultimately help identify optimized treatment modalities based on cumulative data from a multitude of practices and practitioners, which can help surgeons more accurately predict outcomes for their patients.

In current literature and practice, however, there is great heterogeneity in the ideal surgical decision-making strategy based on training, clinical experience, indications, and clinical outcomes [26]. EBM guidelines such as those established by NASS have served to address this challenge and now allow an opportunity for AI and deep learning models to develop an evidence-based understanding of medicine with subsequent utility in clinical settings, perhaps in the future serving as a proxy or mediator for the combined pool of guidelines for any given clinical diagnosis. Our findings demonstrating greater adherence to NASS guidelines for lumbar fusion surgery suggest that, with further clinical testing, LLMs such as GPT-4 may augment clinical decision-making within spine surgery practices. However, this by no means absolves surgeons from the inherent responsibility of utilizing their most informed clinical judgment to ultimately determine indications for surgery.

There are prior studies similarly investigating ChatGPT adherence with clinical guidelines. GPT-3.5 previously demonstrated significant alignment with NASS EBM guidelines for lumbar spine stenosis in a study by Rajjoub et al. [27]. In their investigation, fourteen questions were sourced directly from NASS guidelines and evaluated for agreement with ChatGPT responses. Utilizing a similar methodology, Ahmed et al found GPT-3.5 to have a relatively low accuracy (36.8%-66.7%) referenced to NASS guidelines for degenerative spondylolisthesis, while Mejia et al found GPT-4 to have relatively low accuracy (59%) with reference to NASS clinical guidelines for lumbar disc herniation with radiculopathy [17,20]. These important findings do not align with those of our study, though our investigation is distinguished from those prior in several ways. We utilized well-validated, deidentified clinical vignettes sourced from real spine surgery practices, which further mitigate the potential for leading information or triggers to influence software decision-making. Furthermore, we evaluated the reliability of GPT-4 through several iterations of queries under identical conditions with bias limited from incognito search/investigator blinding and significant internal agreement among queries, which is an important consideration when contemplating how the software may behave in real-time application. Additionally, in contrast, evaluation of accuracy in our study was based on binary responses. While most prior studies have investigated GPT-3.5, this software is no longer available for use whereas GPT-4 is available without any barrier, therefore enhancing the current study's timeliness, availability, and application to real practice. Our study also establishes a statistically significant concordance in decision-making between NASS guidelines for lumbar fusion and GPT-4, which has not been evaluated previously. Finally, given the unique source of our clinical vignettes, we were able to compare GPT-4 responses with those of prior surveyed spine surgeons. Together, these factors strengthen the proposed basis for potential practical application in real clinical scenarios.

### Reliability of GPT-4 decisions

An important factor to consider when evaluating the utility of GPT-4 as an adjunct tool is “interquery reliability”, comparable to inter-rater reliability when evaluating the variability of results between human respondents in identical scenarios. We found that, though in four cases GPT-4 demonstrated instances of internal disagreement, there was a substantial overall internal agreement among the three identical queries for each case. Studies have shown that current medical literature is often conflicted in the correct course of management for patients who may or may not need spinal fusion surgery [26]. Therefore, it is not surprising that the decisions of GPT-4 occasionally conflict within the confines of its own database, which heavily rely on the sum of current literature. Therefore, users should be mindful that while GPT-4 has high reliability based on interquery analysis of indicating necessity for spinal fusion, it may face occasional clinical scenarios in which it cannot provide a stable, definitive answer and these are likely scenarios that are both vague and conflicted between different guidelines. Care should be taken to identify this limitation when applicable, perhaps by implementing a common practice of serial queries and ensuring that provided information is thorough enough for ChatGPT to give appropriately informed responses. Additionally, GPT-4 has limited contextual memory within the parameters of each individual conversation. In other words, while the software largely relies on background offline training for its knowledge base, it has capacity to learn generatively as well. This capacity was not investigated in our research study because the purpose was to better gauge GPT-4’s baseline cognition. However, this can be explored in future studies that would like to determine whether, for example, a spine surgeon can shape the model to adhere to specific preferences in lumbar spine fusion management.

ChatGPT is consistently updated with iterations that aim to improve efficiency, accuracy, and reliability of generated responses [14]. Newer models have been added since the conception of our study, though access to our tested model remain available without paid subscription. Guidelines such as those established by NASS are also subject to change, so this feature lends further credibility to the utility of the software. While our findings are limited to GPT-4, we anticipate that updates – which expand upon existing infrastructure – will lead to further improvements in our evaluated benchmarks.

## Limitations

A limitation of this study is that the NASS “Coverage Policy Recommendations: Lumbar Fusion” was published in 2021, while GPT-4 has been pre-trained using public data from up to 2024. Thus, it is possible for the guidelines to contain relatively outdated information in certain scenarios. We were also restricted to a relatively small sample size of only seventeen cases, with approximately 40% of cases involving the L4-5 levels, thereby limiting the generalizability of our findings. Additionally, due to institutional and logistical limitations, we did not implement or test application in clinical practice. There are many factors that may affect clinical decision-making, so definitive conclusions are limited until utility is tested through clinical application. Though most cases did not involve spinal stenosis, this was the most common pathology (47.1%) which may narrow the scope of application. However, given that the majority of cases were not spinal stenosis, our findings broaden the scope of findings in comparison to prior literature in this realm, which focuses specifically on that diagnosis [27]. Another limitation is that GPT-4 does not have accurate capability of evaluating diagnostic images, so this study was limited to only providing descriptive characteristics of the radiographic, CT, and MRI films. It may be the case that GPT-4’s concordance rate was negatively influenced by its limitation in interpreting images despite having access to the descriptive reports. There have also been several iterations of ChatGPT (e.g. GPT-3.5 prior to GPT-4), therefore the model is continuously updated, and current results may not be generalizable to future software. Another limitation is that the Kappa statistic for interrater reliability testing has a wide margin for agreement among raters. For example, the margin for substantial agreement is 0.61-0.80, meaning that Kappa results just above the 0.61 threshold would still be equivalent in value to 0.80 as substantial agreement [28]. Finally, a notable limitation is the availability of AI models. Generative AI LLMs specifically tuned for medical use such as Med-PaLM, Med-PaLM 2, and MedLM are not publicly available. These are software developed by Google which are showing significantly promising utility for AI-assisted medical decision-making, achieving an accuracy of 85.4% on USMLE-based datasets among other notable benchmarks [29]. This narrowed our study to utilize GPT-4, which is less advanced in this specific regard, but is available to the public with a $20.00 monthly fee or for free with limited access.

## Conclusion

NASS guidelines provide evidence-based suggestions for determining necessity of lumbar spine fusion surgery. GPT-4 adheres to NASS guidelines when evaluating the indications for lumbar spine fusion surgery and may be more adherent to such than board-certified North American spine surgeons. Following EBM guidelines when assessing indications for lumbar fusion surgery leads to improved patient outcomes. Therefore, these findings are promising for the potential role of GPT-4 in supporting decision-making for lumbar fusion surgery, though further research with clinical application and outcomes-based analysis is warranted.

## Declaration of competing interest

The authors declare that they have no known competing financial interests or personal relationships that could have appeared to influence the work reported in this paper.

## Appendix Supplementary materials

Image, application 1
