# Supplementary material for: Evaluation of GPT-4 concordance with north American spine society guidelines for lumbar fusion surgery
Source: N Am Spine Soc J. 2024 Dec 27;21:100580. doi: 10.1016/j.xnsj.2024.100580 (PMC11795085; doi:10.1016/j.xnsj.2024.100580)
Supplement: Supplementary file 1 [file mmc1.docx]

**Supplementary material 1.** 18 items with clinical vignettes and radiological images.

Q1- 75M with prior L4-5 Fusion 10y ago; presents with chronic low back pain and more recent RLE radiculopathy. Unable to walk more than 5min, relieved by sitting  PT, ESI, chiropractor without lasting relief. Str/sens/gait/reflexes normal


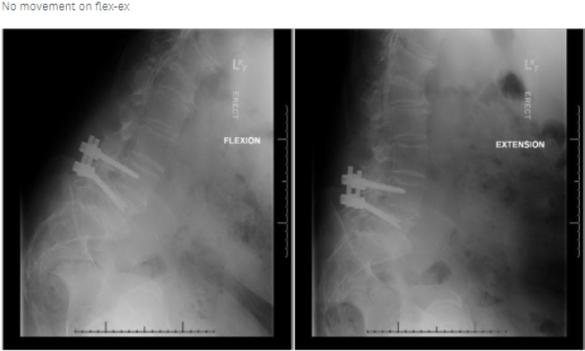
 
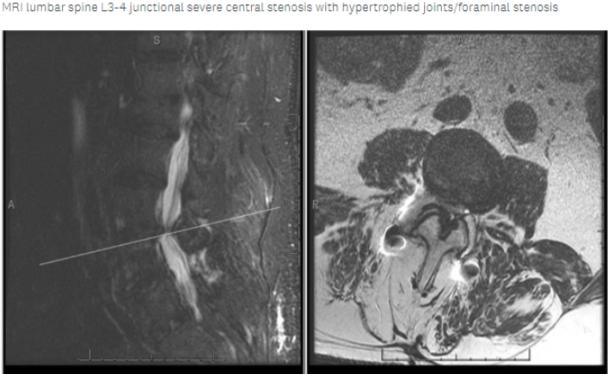

Is fusion surgery indicated for this patient?

- Yes
- No

Q2- 41yo M h/o L4-5 laminectomy/discectomy for intractable R L5 radiculopathy. Returns 3 weeks post-op with a severe back spasm, bilateral LE posterior thigh pain, mechanical back pain. CT scan no bone destruction ESR 73, CRP 10.1 No medical mgmt.

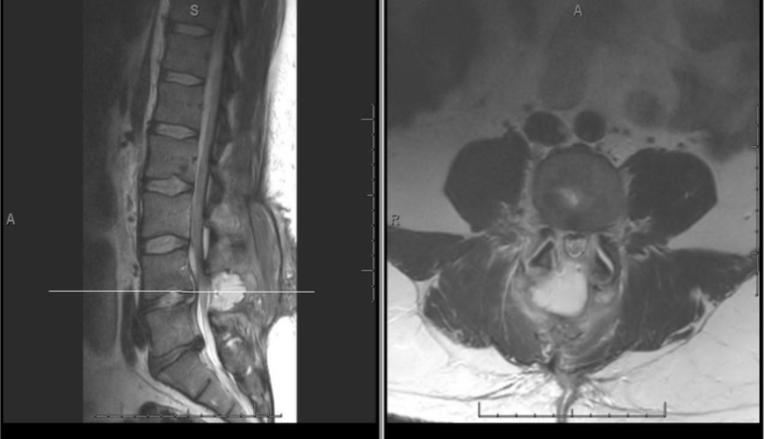


Is fusion surgery indicated for this patient?

- Yes
- No

Q3- 45-year-old female 3 yrs of intractable LBP, unable to walk community distances due to pain. Oswestry Disability Index Score: 36%, which represents moderate disability. Has tried all non-op modalities, No tobacco, No Pysch issues

Single level L5-S1 DJD with disc collapse, modic type I changes in VBs


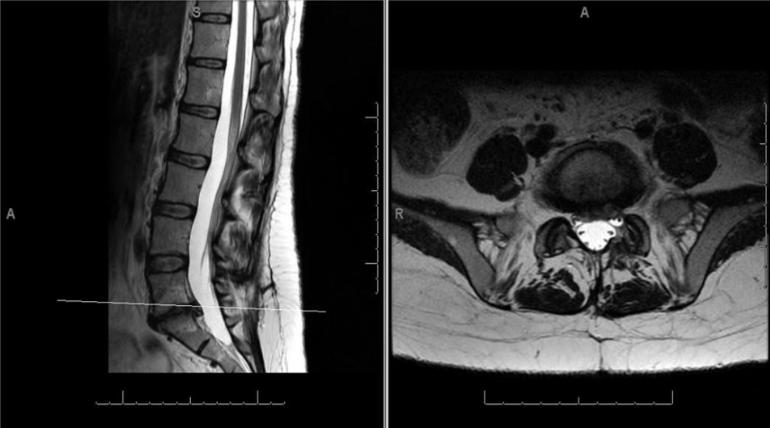


Is fusion surgery indicated for this patient?

- Yes
- No

Q4- 83-year-old female presents with claudicant symptoms requiring rest after 1 block of ambulation.  Denies low back pain; complains of bilateral buttock and thigh pain. Normal exam; No movement on flex-ex

MRI and plain films shows L4-5 deg spondylolisthesis and severe stenosis L2-5. No scoliosis, fracture or instability (except L4-5)


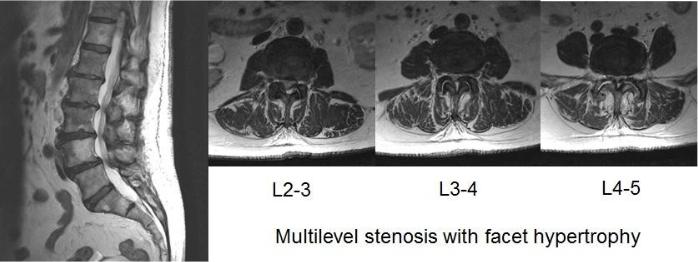


Is fusion surgery indicated for this patient ? If you fuse, how many levels will you need to fuse?

- No
- Yes, 1 level
- Yes, 2 levels
- Yes, 3 or more levels

Q5-68-year-old female severe incapacitating R L5 radiculopathy RLE pain down R buttock, thigh, lateral shin, dorsum of the foot. No improvement with nonoperative strategy x6mo 4+/5 strength in REHL

Plain films - R focal scoliosis L5-S1 with the collapse of the foramen


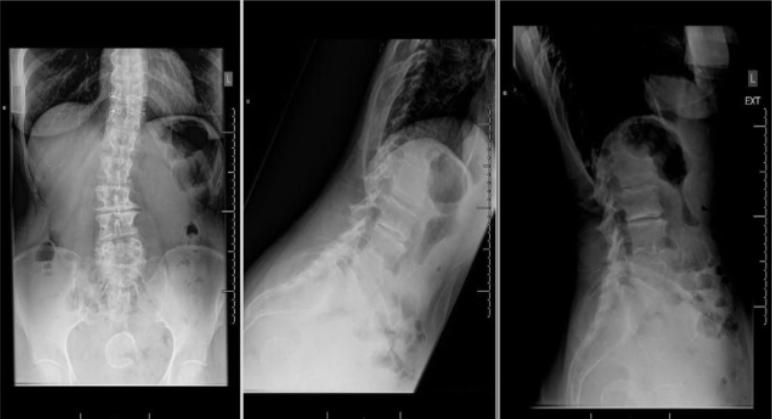


MRI - Severe R L5 foraminal stenosis - lateral disc with complete collapse and obliteration of the foramen

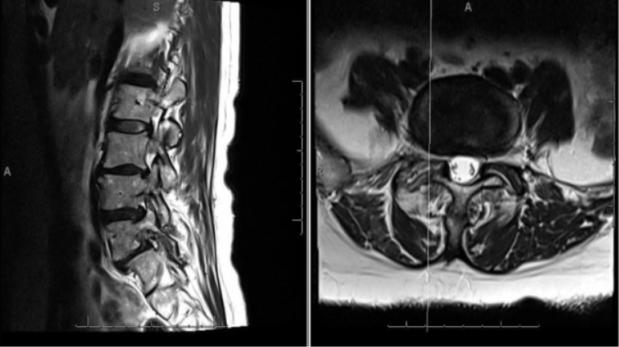


Is fusion surgery indicated for this patient ?

- Yes
- No

Q6- 60-year-old female had prior L4-5 laminectomy for spinal stenosis 8y ago. Presents with recurrent R>L LE pain down lateral aspect of leg to R foot. Symptoms improved with sitting/laying down. ESI with decreasing relief Normal exam   

MRI-Bilateral facet hypertrophy with foraminal sever recurrent stenosis L4-5 at area of previous decompression


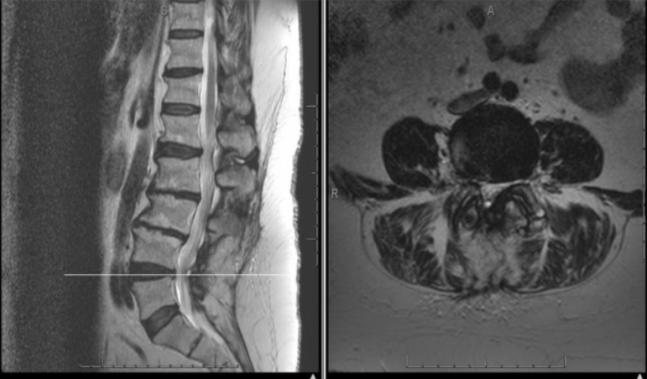

CT Scan prior laminectomy defect with bilateral facet hypertrophy and foraminal narrowing

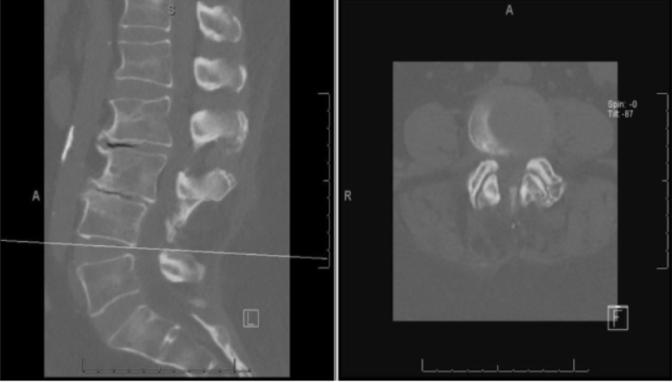


Is fusion surgery indicated for this patient?

- Yes
- No

Q7- 58-year-old M with h/o third disc herniation at L5-S1 within 3 yrs. Now with transient improvement after the last operation with recurrence after 9 months. Refractory to medical management. No back pain. Normal examination. No instability on flex-ex 

MRI Lumbar - Recurrent L5-S1 disc herniation after microdiscetomy


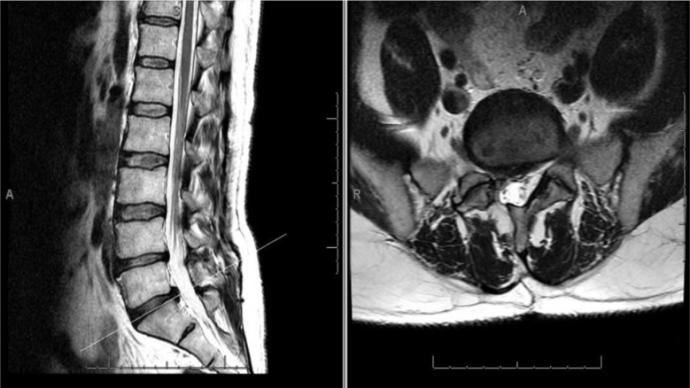


Is fusion surgery indicated for this patient?

- Yes
- No

Q8- 72-year-old healthy male who presents with 3mo R L4 and L5 radiculopathy. Minimal low back pain. ESIx2 with improvement for 2 wks. No movement on flexion/extension. Normal exam.  Plain films - R focal scoliosis L5-S1 with the collapse of the foramen
MRI L4-5 degenerative spondylolisthesis and severe bilateral lateral recess stenosis at L4-5


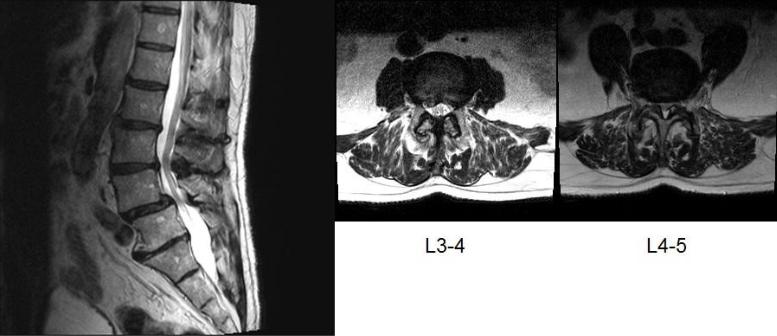


Is fusion surgery indicated for this patient?

- Yes
- No

Q9- 50-year-old male h/o morbid obesity presents with severe axial back pain, relieved with supine positioning, worse when upright. Long-standing, but worse over the past 4 weeks.  Normal strength/sensation/gait/reflexes . No instability on flex-ex

MRI - Multilevel degenerative changes; Worse L2-4 and not scoliosis or deformity


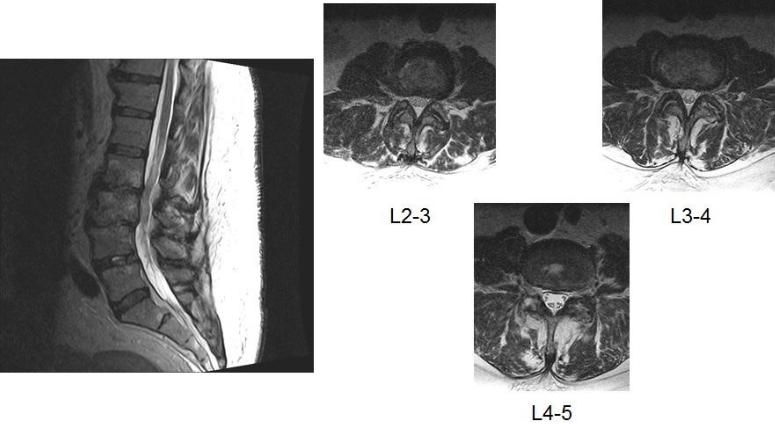


Is fusion surgery indicated for this patient?

- Yes
- No

Q10- 58-year-old female with 6mo h/o L5 radiculopathy. ESI provided 1 week of relief. No low back pain. Normal physical exam. No instability on flex-ex

MRI - No anterolisthesis at L4-5 with large L4-5 synovial cyst


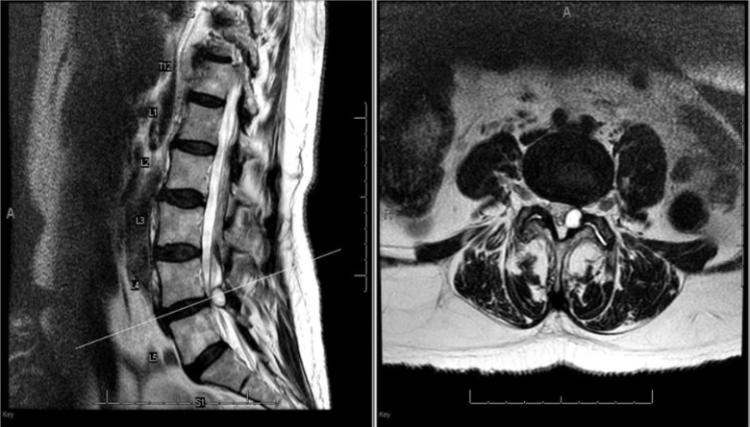

Is fusion surgery indicated for this patient?

- Yes
- No

Q11- 61M h/o L4-S1 decompression and fusion 18mo for lumbar stenosis. Improved for 6mo, presents with worsening mechanical back pain, worse when standing. Requiring increasing doses of pain medication. Normal strength/sensation/gait/reflexes


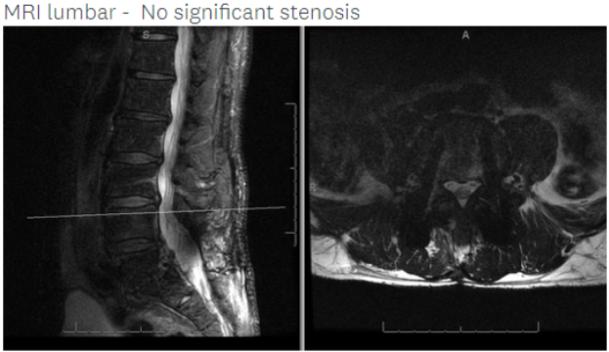


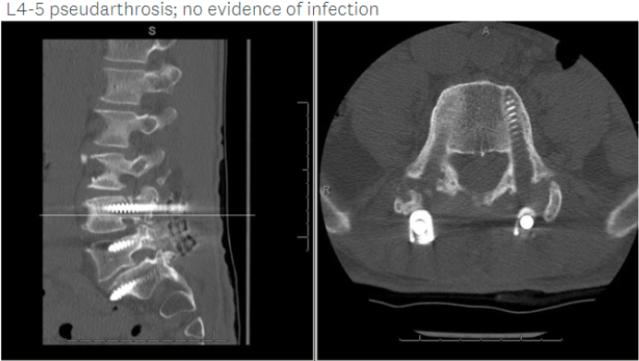


Is fusion surgery indicated for this patient?

- Yes
- No

Q12- 75-year-old male with chronic back and bilateral buttock pain. Non-operative strategies tried included: 2 years of physical therapy, ESI, Chiropractor and SCS with no assistance. Neurogenic claudication, unable to ambulate more than 100 feet


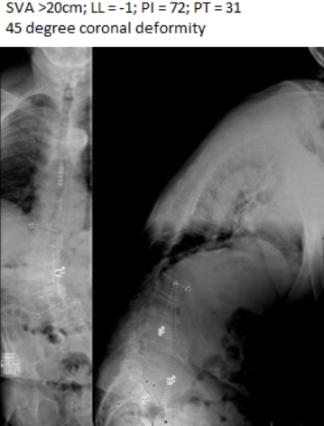


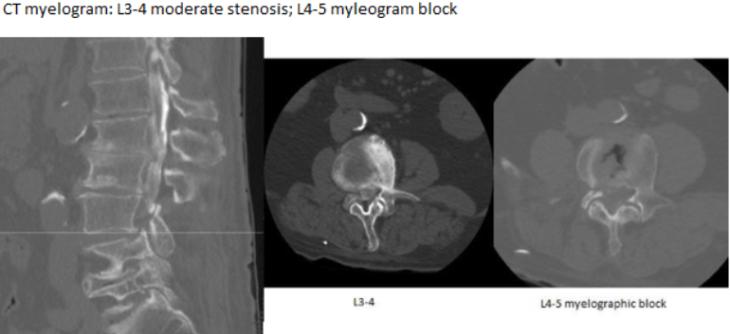


Is fusion surgery indicated for this patient?

- Yes
- No

Q13- 22 M s/p fall from the 4th story window. Complains of severe immediate low back pain. Neurologically intact.


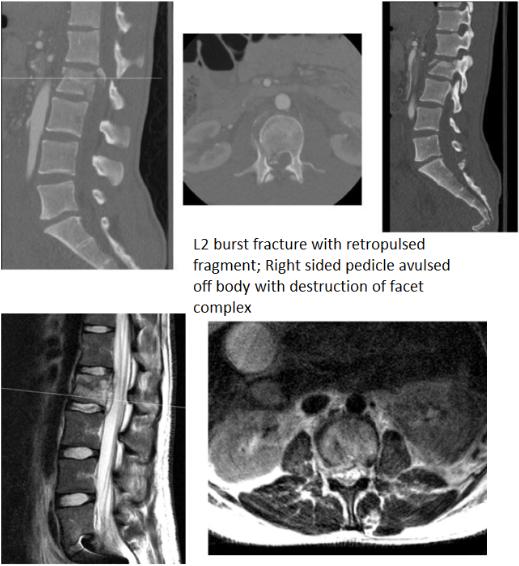


 
Is fusion surgery indicated for this patient?

- Yes
- No

Q14- 53M with 2mo RLE pain began acutely after a skiing trip. Progressive pain/weakness in the quad, pain in the anterior thigh. Difficulty walking up steps  4-/5 weakness in R quad. No instability on flex-ex.


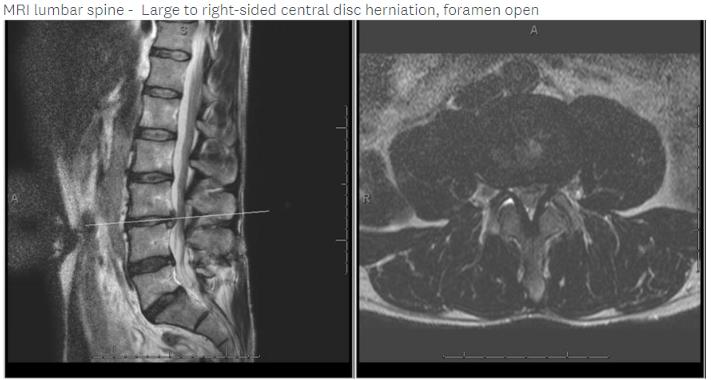


  Is fusion surgery indicated for this patient ?

- Yes
- No

Q15- 60M complains of bilateral buttock/LE pain for several years, worse over the past 3-4 months, causing limited activity, unable to walk >1 city block without stopping to sit Transient relief with ESI, worsening pain with PT. Normal exam


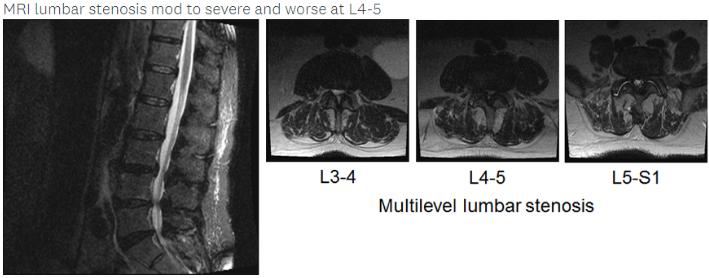


  Is fusion surgery indicated for this patient?

- Yes
- No

Q16- 63F s/p fall downstairs complains of low back pain. Normal neuro exam.

    
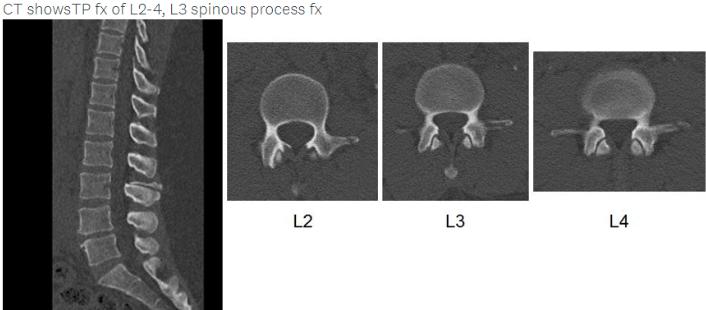


  Is fusion surgery indicated for this patient?

- Yes
- No

Q17- 75M with back and bilateral buttock pain for 1 month. Non-operative strategies tried included: ESI, Chiropractor and SCS with no assistance. Neurogenic claudication, unable to ambulate more than 100 feet


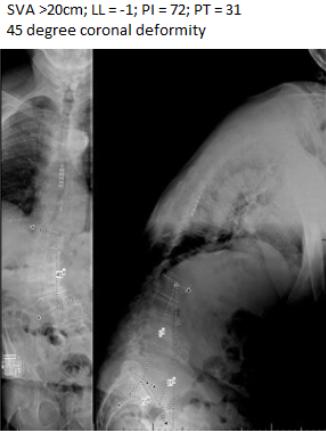


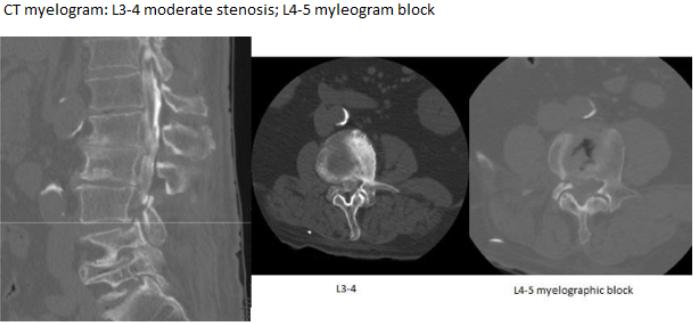

   Is fusion surgery indicated for this patient?

- Yes
- No

Q18- 32F with low back pain which worsened recently after she slid down her carpeted steps. Normal neuro exam. Non-operative care for 6 weeks and positive tobacco user.


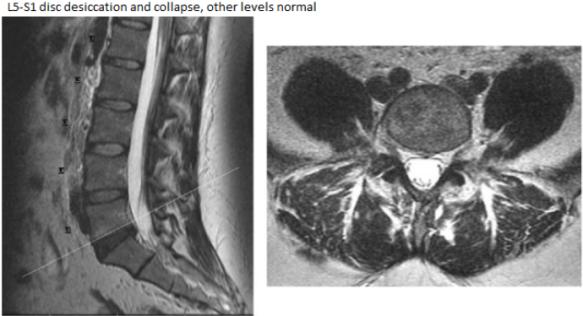


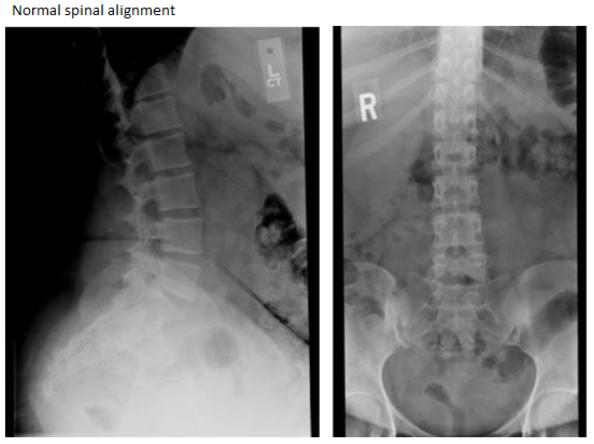


Is fusion surgery indicated for this patient?

- Yes
- No
